# Neuroactive Steroids Reverse Tonic Inhibitory Deficits in Fragile X Syndrome Mouse Model

**DOI:** 10.3389/fnmol.2019.00015

**Published:** 2019-02-05

**Authors:** Amit Modgil, Thuy N. Vien, Michael A. Ackley, James J. Doherty, Stephen J. Moss, Paul A. Davies

**Affiliations:** ^1^Department of Neuroscience, Tufts University School of Medicine, Boston, MA, United States; ^2^Sage Therapeutics, Inc., Cambridge, MA, United States; ^3^Department of Neuroscience, Physiology and Pharmacology, University College, London, United Kingdom

**Keywords:** GABA_A_ receptor (GABA_A_R), tonic inhibition, neurosteroid, fragile X, phosphorylation, benzodiazepine

## Abstract

Fragile X syndrome (FXS) is the most common form of inherited intellectual disability. A reduction in neuronal inhibition mediated by γ-aminobutyric acid type A receptors (GABA_A_Rs) has been implicated in the pathophysiology of FXS. Neuroactive steroids (NASs) are known allosteric modulators of GABA_A_R channel function, but recent studies from our laboratory have revealed that NASs also exert persistent metabotropic effects on the efficacy of tonic inhibition by increasing the protein kinase C (PKC)-mediated phosphorylation of the α4 and β3 subunits which increase the membrane expression and boosts tonic inhibition. We have assessed the GABAergic signaling in the hippocampus of fragile X mental retardation protein (FMRP) knock-out (*Fmr1* *KO*) mouse. The GABAergic tonic current in dentate gyrus granule cells (DGGCs) from 3- to 5-week-old (p21–35) *Fmr1* *KO* mice was significantly reduced compared to WT mice. Additionally, spontaneous inhibitory post synaptic inhibitory current (sIPSC) amplitudes were increased in DGGCs from *Fmr1 KO* mice. While sIPSCs decay in both genotypes was prolonged by the prototypic benzodiazepine diazepam, those in *Frm1-KO* mice were selectively potentiated by RO15-4513. Consistent with this altered pharmacology, modifications in the expression levels and phosphorylation of receptor GABA_A_R subtypes that mediate tonic inhibition were seen in *Fmr1 KO* mice. Significantly, exposure to NASs induced a sustained elevation in tonic current in *Fmr1 KO* mice which was prevented with PKC inhibition. Likewise, exposure reduced elevated membrane excitability seen in the mutant mice. Collectively, our results suggest that NAS act to reverse the deficits of tonic inhibition seen in FXS, and thereby reduce aberrant neuronal hyperexcitability seen in this disorder.

## Introduction

Fragile X syndrome (FXS) is the most common form of inherited intellectual disability and a major cause of autism spectrum disorders (ASDs) yet there are limited treatments to limit the impact of FXS and other ASDs. In addition to the intellectual disability there is increased incidence of hyperactivity, sleep irregularities, and seizure activity seen in FXS. The underlying cause of FXS is a loss of the fragile X mental retardation protein (FMRP; Verkerk et al., 1991). Loss of FMRP expression leads to hyperexcitability that underlie the varying deficits in FXS. Studies from both FXS patients and animal models have revealed altered expression levels of γ-aminobutyric acid type A receptor (GABA_A_R) α4/δ subunits with a concomitant reduced efficacy of tonic inhibition, a non-synaptic type of inhibition important for regulating the excitability of neurons and the activity of neuronal circuits (D’Hulst et al., 2006; Belelli et al., 2009; Curia et al., 2009; Fatemi et al., 2009, 2010; Zhang et al., 2017). The mechanism that results in this deficit of tonic inhibition remains unknown as little is known on how the membrane trafficking of α4 subunit containing GABA_A_Rs is regulated and if deficits in these processes contribute to FXS.

α4 subunit containing GABA_A_Rs are located primarily extrasynaptically and are persistently activated by ambient concentrations of GABA (Glykys and Mody, 2007). Extrasynaptic GABA_A_Rs are responsible for mediating tonic inhibition that determines the gain and offset of the neuronal output, thus regulating the excitability of neurons and the activity of neuronal circuits (Semyanov et al., 2004; Belelli et al., 2009). Expression of α4 subunit containing GABA_A_Rs dramatically increases during a “critical postnatal period” of hippocampal and cortical development (LeBlanc and Fagiolini, 2011; Meredith et al., 2012). Studies from patients and mouse models of FXS have revealed alterations in the expression levels of GABA_A_R α4, β, and δ subunits during this critical period with a concomitant reduction in the efficacy of tonic inhibition (D’Hulst et al., 2006; Gantois et al., 2006; Curia et al., 2009). Deficits in the activity of α4 subunit-containing GABA_A_Rs are implicated in other ASDs, sleep disturbances and epilepsy (Belelli et al., 2009; Brickley and Mody, 2012; Deidda et al., 2014). Additionally, modifications in GABA_A_R β3 subunit gene, and/or protein levels are implicated as causes of epilepsy, and ASD (Delahanty et al., 2011; Kang and Barnes, 2013). Phosphorylation of serine residues 408 and 409 (S408/9) in the β3 subunit reduces the endocytosis of GABA_A_Rs from the plasma membrane, an effect that is mimicked by their mutation to alanines. We created a mouse in which S408/9 have been mutated to alanines (S408/9A). S408/9A mice exhibited a decreased tonic but increased phasic inhibition, altered dendritic spine structure, increased repetitive behavior, decreased social interaction, and an epileptic phenotype; replicating core ASD phenotypes (Vien et al., 2015).

The neurobiology and circuit abnormalities of the dentate gyrus (DG) in ASD have not been extensively studied despite the important role played by the DG in many aspects of ASD pathophysiology. The DG is important for encoding information for the hippocampus and is especially important for episodic memory which is impaired in ASD (Lind, 2010; Rangel et al., 2014). A co-morbidity of ASD with EEG abnormalities and seizures has been reported (ranges as high as 40% in some studies) and those children with ASD and seizures have poorer cognitive behavior than children with either just ASD or epilepsy (Chez et al., 2006; Canitano, 2007; Hara, 2007). Adolescents and young adults with ASD, FXS, and intellectual disability have a greater rate of hospitalization for epilepsy than the general population (McDermott et al., 2015). FXS patients have sleep disturbances and seizures in these patients occur more frequently during sleep (Musumeci et al., 1990, 1999). The root cause of these co-morbid conditions is unknown but deficits in GABAergic signaling; particularly tonic inhibition has been suggested (Bozzi et al., 2018). The DG is a major site for epileptogenesis occurring during the breakdown of the dentate gate, a filter for the incoming perforant neuronal pathway which protects the hippocampus from excessive neuronal activity and excitotoxic damage (Goldberg and Coulter, 2013).

## Materials and Methods

### Animals

*Fmr1-KO* mice were originally purchased from The Jackson Laboratory (B6.129P2-*Fmr1*^tm1Cgr^/J) and then bred in house (homozygous female × hemizygous male). Fmr1-KO and WT C57BL/6 mice were housed under constant temperature and humidity on a 12-h light/dark cycle with standard rodent food and water *ad libitum*. Male mice were used in the current study. This study was carried out in accordance with the recommendations of The Institutional Animal Care and Use Committee of Tufts University & Tufts Medical Center. The protocol was approved by the The Institutional Animal Care and Use Committee of Tufts University & Tufts Medical Center.

### Hippocampal Slice Preparation

Brain slices were prepared from 3- to 5-week-old male C57 mice. Mice were anesthetized with isoflurane, decapitated, and brains were rapidly removed and submerged in ice-cold cutting solution containing (mM): 126 NaCl, 2.5 KCl, 0.5 CaCl_2_, 2 MgCl_2_, 26 NaHCO_3_, 1.25 NaH_2_PO_4_, 10 glucose, 1.5 sodium pyruvate, and 3 kynurenic acid. Coronal 310 μM thick slices were cut with the vibratome VT1000S (Leica Microsystems, St Louis, MO, USA). The slices were then transferred into incubation chamber filled with prewarmed (31–32°C) oxygenated artificial cerebro-spinal fluid (ACSF) of the following composition (in mM): 126 NaCl, 2.5 KCl, 2 CaCl_2_, 2 MgCl_2_, 26 NaHCO_3_, 1.25 NaH_2_PO_4_, 10 glucose, 1.5 sodium pyruvate, 1 glutamine, 3 kynurenic acid and 0.005 GABA bubbled with 95% O_2_-5% CO_2_. Exogenous GABA was added in an attempt to standardize ambient GABA in the slice and provide an agonist source for newly inserted extrasynaptic GABA_A_Rs. Slices were allowed to recover at 32°C for at least 30 min before exposure to neuroactive steroids (NASs) or vehicle.

### Neuroactive Steroid Incubation

Hippocampal slices were incubated for 15 min in a chamber containing either vehicle control or NASs dissolved in ACSF that did not contain kynurenic acid. Following this incubation, slices were transferred to a submerged, dual perfusion recording chamber (Warner Instruments, Hamden, CT, USA) on the stage of an upright microscope (Nikon FN-1) with a 40× water immersion objective equipped with DIC/IR optics. Slices were maintained at 32°C and gravity-superfused with ACSF solution (with kynurenic acid) throughout experimentation and perfused at rate of 2 ml/min with oxygenated (O_2_/CO_2_ 95/5%) ACSF. Slices were perfused for 30 to 60 min before recordings were started.

### Electrophysiology Recordings

Whole-cell currents were recorded from the DG granule cells (DGGCs) in 310-μM-thick coronal hippocampal slices. Patch pipettes (5–7 MΩ) were pulled from borosilicate glass (World Precision Instruments) and filled with intracellular solution of the composition (in mM) as follows: 140 CsCl, 1 MgCl_2_, 0.1 EGTA, 10 HEPES, 2 Mg-ATP, 4 NaCl and 0.3 Na-GTP (pH = 7.2 with CsOH). For action potential recordings in current-clamp mode, the intracellular solution consisted of (in mM): 145 K-gluconate, 5 NaCl, 10 HEPES, 0.1 EGTA, 2 Mg-ATP, and 0.3 Na-GTP (pH = 7.2 with CsOH).

A 5-min period for stabilization after obtaining the whole-cell recording conformation (holding potential of −60 mV) was allowed before currents were recorded using an Axopatch 200B amplifier (Molecular Devices), low-pass filtered at 2 kHz, digitized at 20 kHz (Digidata 1440A; Molecular Devices), and stored for off-line analysis. The liquid junction potential error of 15 mV was arithmetically corrected in all current-clamp recording data.

### Electrophysiology Analysis

For tonic current measurements, an all-points histogram was plotted for a 10-s period before and during 100 μM picrotoxin (PTX) application, once the response reached a plateau level. Recordings with unstable baselines were discarded. Fitting the histogram with a Gaussian distribution gave the mean baseline current amplitude and the difference between the amplitudes before and during PTX was considered to be the tonic current. The negative section of the all-points histogram which corresponds to the inward inhibitory post synaptic inhibitory currents (IPSCs) was not fitted with a Gaussian distribution (Nusser and Mody, 2002; Kretschmannova et al., 2013). Series resistance and whole-cell capacitance were continually monitored and compensated throughout the course of the experiment. Recordings were eliminated from data analysis if series resistance increased by >20%.

Spontaneous IPSCs (sIPSCs) were analyzed using the mini-analysis software (version 5.6.4; Synaptosoft, Decatur, GA, USA). Minimum threshold detection was set to three times the value of baseline noise signal. To assess sIPSC kinetics, the recording trace was visually inspected and only events with a stable baseline, sharp rising phase, and single peak were used to negate artifacts due to event summation. Only recordings with a minimum of 200 events fitting these criteria were analyzed. sIPSCs amplitude, and frequency from each experimental condition was pooled and expressed as mean ± SEM. To measure sIPSC decay we averaged 100 consecutive events and fitted the decay to a double exponential and took the weighted decay constant (τ_w_). Statistical analysis was performed by using Student *t*-test (paired and unpaired where appropriate), where *p* < 0.05 is considered significant.

To measure the excitability of DGGCs action potentials were elicited with depolarizing square current injections varying between 20 and 300 pA for 500 ms. Input-output curves were plotted as the total number of AP spikes fired vs. the current injection for both WT and *Fmr1* KO. The AP properties were statistically compared between groups.

### Western Blotting

Standard Western blotting protocols were performed as described previously (Vien et al., 2015). The hippocampus from from both genotypes were rapidly dissected, flash-frozen, and lysed in lysis buffer composed of (in mM): 20 Tris·HCl (pH 8.0), 150 NaCl, 1% Triton X-100, 5 EDTA, 10 NaF, 2 Na_3_VO_4_, 10 pyrophosphate, and 0.1% SDS. Total protein concentration was established, and 40 μg of hippocampal lysate was subjected to SDS/PAGE, transferred to nitrocellulose membranes, and blocked with 5% (wt/vol) BSA in Tris-buffered saline-Tween 20 for 1 h. Membranes were immunoblotted with the indicated primary antibodies, and following extensive rinsing, they were probed with HRP-conjugated secondary antibodies and detected with enhanced chemiluminescence. Blots were imaged, and data were normalized to actin and quantified with the CCD-based LAS 3000 system (FujiFilm).The antibodies against the GABA_A_R α1, α2, α4, β1, β2, β3 and γ2 subunits were purchased from Neuromab. The phospho specific antibody produced against S443 (pS443), was raised in rabbits against a synthetic peptide derived from the murine α4 subunit in which S443 was phosphorylated (PGSLGSASTRPA). For the β3 subunit, samples were blotted with pS408/9 and β3 subunit antibodies as detailed previously (Jovanovic et al., 2004). The ratios of pS443/α4 and pS408/9/β3 subunit immunoreactivity were compared between genotypes. We also examined the phosphorylation of S383 in the β3 subunit, which is a substrate of CamKII, but not PKC, using the respective phospho-specific antibody pS383 (Saliba et al., 2012).

## Results

### Tonic Inhibition Is Reduced in DGGCs of *Fmr1 KO* Mice

**Figure 1:**
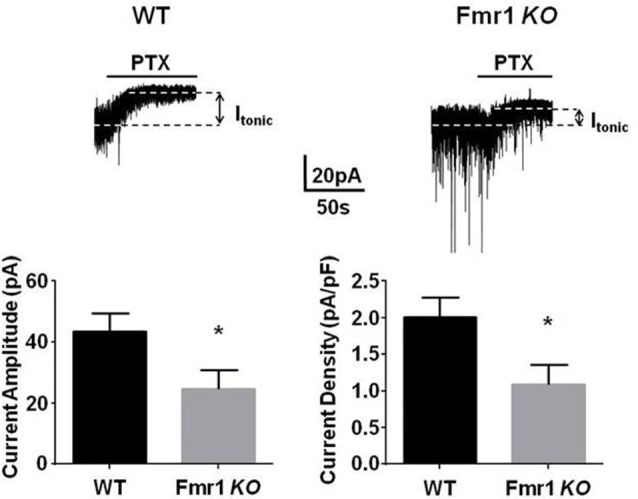
*Fmr1 KO* mice display deficits in tonic current. Recordings were made from dentate gyrus granule cells (DGGCs) in hippocampal slices from p21 to 35 WT C57 controls, or *Fmr1 KO* mice in the presence of 5 μM γ-aminobutyric acid (GABA). Tonic current was determined by measuring the difference in holding current amplitude before and after applying 100 μM picrotoxin (PTX). *Fmr1 KO* mice exhibited a significant reduction in tonic current amplitude. *Significantly different to WT control, see text for *p* value (*t*-test, *n* = 10–12 cells).

α4/δ subunit containing GABA_A_Rs, which mediate tonic inhibition, are highly expressed in the DG. However, to date it remains unclear if the efficacy of tonic inhibition is altered within this key structure in FXS. To directly test this, we compared the tonic current in DGGCs from *Fmr1 KO* mice on the C57/BL6 background and WT controls. Hippocampal slices were prepared from 3- to 5-week old mice and tonic current were measured using patch clamp recordings. Tonic currents were measured as the change in baseline amplitude in the presence of 5 μM GABA, alone, and in the presence of the GABA_A_ receptor antagonist PTX. We noted that there was a significant decrease in tonic current in *Fmr1 KO* mice compared to controls (~50%; *p* = 0.015). This reduction was not due to smaller neurons in *Fmr1 KO* mice as the current amplitude was normalized to membrane capacitance and the resulting current density showed an identical reduction (Figure 1).

### The Efficacy of Phasic Inhibition of *Fmr1 KO* Mice

**Figure 2:**
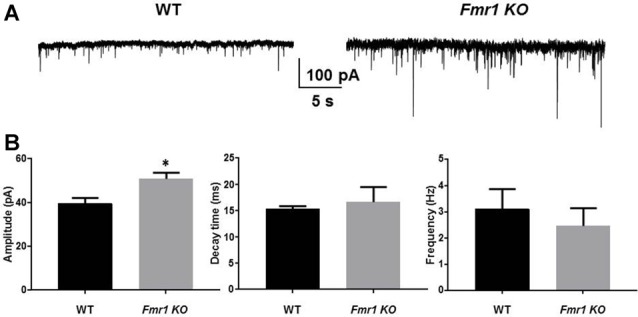
*Fmr1 KO* mice display alterations in phasic current. Recordings were made from DGGCs in hippocampal slices from p21-35 WT, or *Fmr1 KO* mice **(A)**. *Fmr1 KO* mice displayed larger spontaneous inhibitory post synaptic currents (sIPSC) amplitudes compared to age-matched controls as seen in **(B)** mean sIPSC amplitude, decay, and frequency from WT and *Fmr1 KO* mice (*significantly different to WT controls, see text for *p* value; *t*-test, from seven slices).

In autism, benzodiazepines can have paradoxical behavioral responses indicating an altered phasic inhibitory response (Marrosu et al., 1987; Tranfaglia, 2011). We recorded sIPSCs from DGGCs in *Fmr1 KO* and WT mice. There was a significant increase in the amplitude of sIPSCs in *Fmr1 KO* mice (51 ± 3.1 pA, *n* = 7) compared to WT mice (39.7 ± 2.5 pA, *n* = 6; *p* = 0.02). No change in sIPSC decay in DGGCs from *Fmr1 KO* mice (WT, 15.4 ± 0.6 ms, *n* = 6; *Fmr1 KO*, 16.7 ± 2.8 ms, *n* = 7; *p* = 0.7), nor IPSC frequency (WT, 3.1 ± 0.8 Hz, *n* = 6; *Fmr1 KO*, 2.5 ± 0.7 Hz, *n* = 7; *p* = 0.5) was detected (Figure 2). These data would suggest that there is a drastic change in inhibitory drive in the DG of *Fmr1 KO* mice. To explore this further we examined the expression of GABA_A_R subunits.

### Synaptic Targeting of α4 Subunit Containing GABA_A_Rs in *Fmr1 KO* Mice

**Figure 3:**
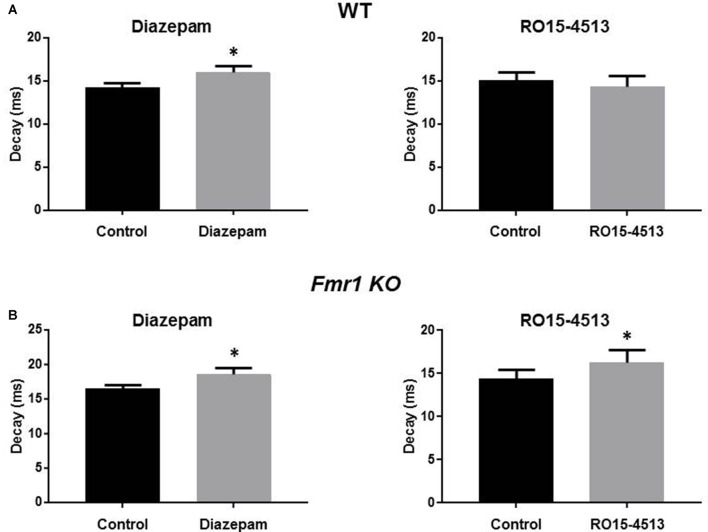
Modulation of DGGC IPSCs by benzodiazepines. Recordings were made from DGGCs in hippocampal slices from p21-35 WT, or *Fmr1 KO* mice. Bar graphs show the effects of acute (10 min) exposures to diazepam (left column) or Ro15-4513 (right column) applied to slices from **(A)** wild-type (*n* = 6) and **(B)** *Fmr1 KO* (*n* = 7) mice. See text for *p* values.

The alterations in sIPSC properties observed in *Fmr1 KO* mice resemble those seen in previous studies with ethanol exposed mice and a mouse model of epilepsy where the α4 subunit integrates with synaptic GABA_A_Rs (Sun et al., 2007; Olsen and Spigelman, 2012). Therefore, we assessed the effects of the benzodiazepines diazepam (which enhance αβγ2 GABA_A_Rs where α can be 1-3, and 5 subunits) and Ro15-4513, (an inverse agonist at diazepam sensitive receptors but an agonist on α4βγ2 GABA_A_Rs) on sIPSC properties in hippocampal slices from WT and *Fmr1 KO* mice. Diazepam (30 nM) significantly prolonged sIPSC decay in WT (14.3 ± 0.5 ms to 16 ± 0.8 ms, *n* = 6, *p* = 0.02) and *Fmr1 KO* DGGCs (16.5 ± 0.5 ms to 18.6 ±0.9 ms, *n* = 7, *p* = 0.02, Figure 3). No significant changes in sIPSC amplitude or frequency resulted from diazepam exposure in WT or *Fmr1 KO* slices (data not shown). Conversely, in the presence of Ro15-4513 (300 nM), sIPSC amplitude, decay, and frequency were all unchanged in WT slices (data not shown). However, DGGC sIPSC decay was significantly enhanced in slices from *Fmr1 KO* mice (14.4 ± 1.1 ms to 16.3 ± 1.4 ms, *n* = 7, *p* = 0.04, Figure 3). sIPSC amplitude, and frequency were all unchanged in *Fmr1 KO* slices exposed to Ro15-4513 (data not shown). These data suggest an increased contribution of α4 subunit containing GABA_A_Rs to synaptic currents in the DG of FXS mice.

### Young, but Not Older, *Fmr1 KO* Mice Have Alterations in Hippocampal GABA_A_R Subunit Phosphorylation and Expression

To assess if these modifications reflect alterations in the phosphorylation and/or stability of GABA_A_Rs, we compared these parameters in hippocampal slices from both genotypes. We examined the phosphorylation of S443 in the α4 subunit, in addition to S408/9 in the β3 subunit. These residues are accepted substrates of PKC and their phosphorylation plays key roles in regulating the membrane trafficking of GABA_A_Rs. In the case of S443 its phosphorylation acts to increase insertion of α4 subunit containing GABA_A_Rs into the plasma membrane and sustained increase in tonic inhibition. In contrast, phosphorylation of S408/9 acts to reduce receptor endocytosis increasing their accumulation at inhibitory synapses (Abramian et al., 2014, 2010; Adams et al., 2015).

To measure the expression levels of GABA_A_Rs that mediate phasic inhibition, we measured levels of the α1, α2 and γ2 subunit, and for tonic inhibition we employed antibodies against the α4, α5 and δ subunits. The levels of β3 subunit that is a component of subtypes that mediate both forms of GABAergic inhibition was also compared between genotypes.

**Figure 4:**
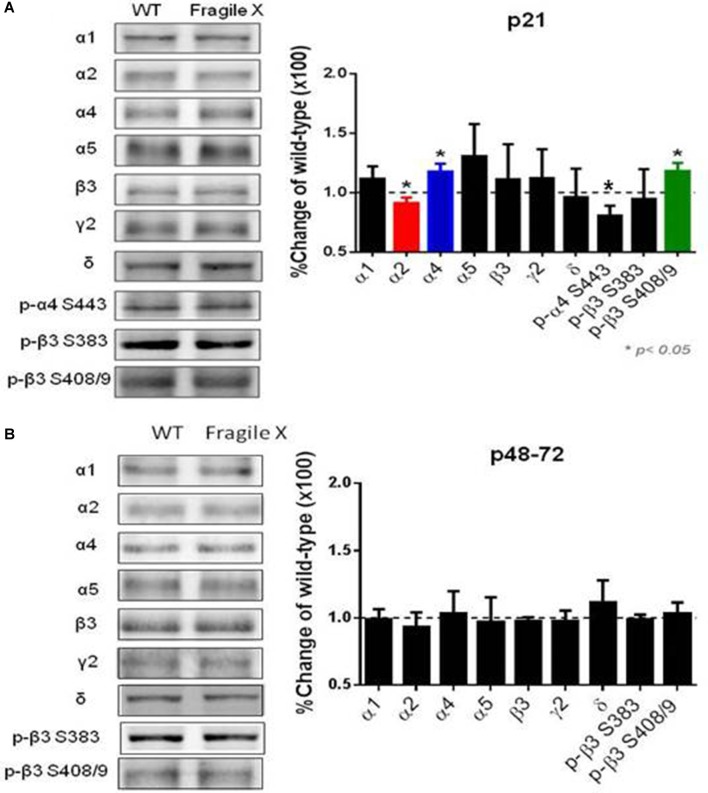
Comparison of GABA_A_R subunit expression and phosphorylation in *Fmr1 KO* mice. Fifty microgram of SDS-soluble hippocampal extracts from **(A)** p21, **(B)** p48-72 C57/BI6 *Fmr1 KO* mice, or WT controls were subject to immunoblotting with antibodies against the GABA_A_R, α1, α2, α4, α5, β3, γ2 and δ. Total subunit expression levels were then normalized to WT. In addition, extracts were immunoblotted with p-α4 S443, p-β3 S408/9 and p-β3 383 antibodies. The ratios of pS409/9/β3 and pS383/β3 immunoreactivity were compared and normalized to values seen in WT mice. *Significantly different to WT controls see text for *p* values (*t*-test; *n* = 3–8 mice).

Using immunoblotting we compared the expression levels and phosphorylation of GABA_A_R subunits in *Fmr1 KO* mice. We measured these parameters in hippocampal extracts from p21 C57 *Fmr1 KO* mice, a developmental time point at which these mice exhibit an accepted seizure phenotype. Immunoblotting revealed that phosphorylation of S443 in the α4 subunit was significantly decreased in *Fmr1 KO* mice 80 ± 9% of control (*n* = 4 for WT and *Fmr1 KO* *p* = 0.001, while that for β3 S408/9 was increase 120 ± 6% of control (*n* = 6 for WT and *Fmr1 KO*; *p* = 0.005). In contrast, phosphorylation of β3 S383 was comparable between genotypes. Interestingly, total α4 subunit levels were increased to 117.5 ± 6.7% of WT (*n* = 3, *p* = 0.026) while those for α2 were decreased to 91 ± 5% of WT (*n* = 5 WT, *n* = 4 *Fmr1 KO*; *P* = 0.04). In contrast to this, the total expression levels of α1, α5, β3, δ and γ2 subunits were comparable between genotypes (Figure 4A). Finally, we examined if these changes in GABA_A_R expression level and phosphorylation persist in adult mice. Significantly, in p48–72 C57 *Fmr1 KO* mice did not exhibit any deficits in GABA_A_R expression levels or phosphorylation (Figure 4B). This experiment demonstrates that there are alterations in the phosphorylation and expression levels of GABA_A_Rs in *Fmr1 KO* mice.

### Neuroactive Steroids Reverse the Deficits in Tonic Inhibition

We have published the ability of the NASs, tetrahydrodeoxycorticosterone (THDOC), ALLO, or the novel synthetic NAS, SGE-516 to increase tonic current through a mechanism that involves protein kinase C (PKC)-mediated phosphorylation S443 of the α4 subunit (Abramian et al., 2014; Modgil et al., 2017). The next stage is to ask whether the deficit in tonic current observed in DGGCs from *Fmr1 KO* mice (Figure 1) could be reversed by NAS treatment. A PKC-mediated increase in tonic inhibition in slices from *Fmr1 KO* mice, as seen in WT slices, could indicate a novel therapeutic mechanism to increase the inhibitory tone in FXS.

**Figure 5:**
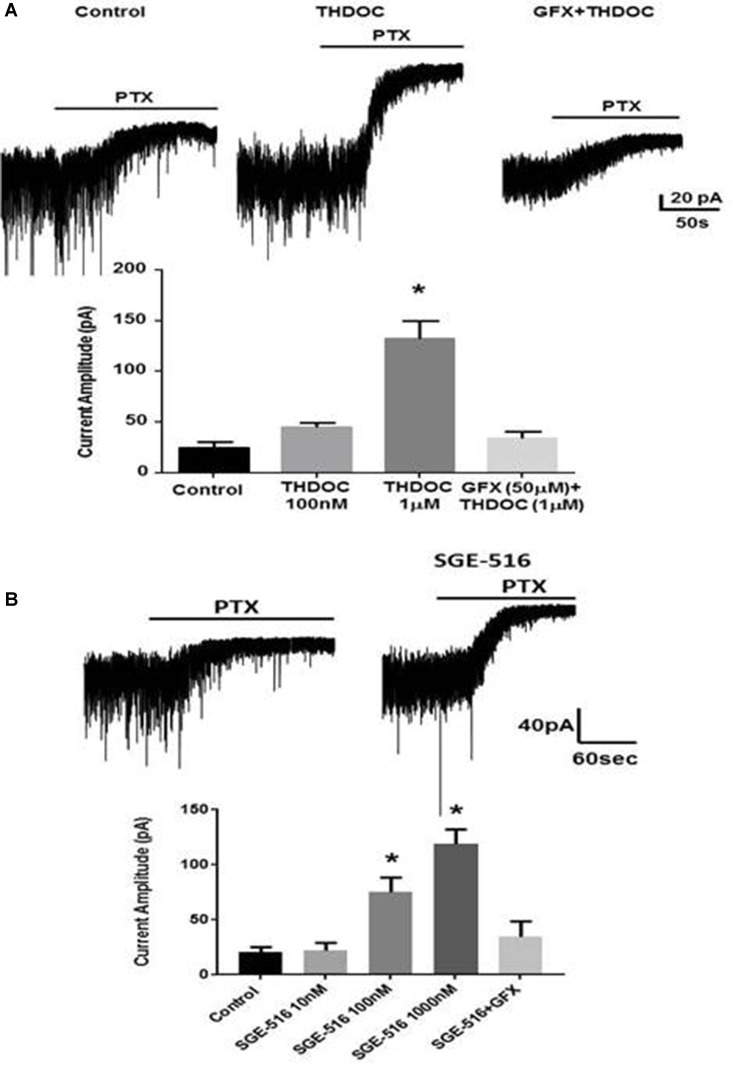
Deficits in tonic inhibition in *Fmr1 KO* mice can be rescued by neuroactive steroids (NASs). Recordings were made from DGCCS in hippocampal slices from p21-35 *Fmr1 KO* mice in the presence of 5 μM GABA followed by 20 μM PTX and the difference in holding was then determined. **(A)** Examples of tonic currents from *Fmr1 KO* mice slices pre-incubated for 15 min with control vehicle, or 1 μM tetrahydrodeoxycorticosterone (THDOC) then washed for >30 min before recordings began. No change in tonic current was observed in slices pre-incubated for 15 min with GFX followed by THDOC. Bottom. Mean data showing 100 nM THDOC slightly increases tonic current whereas 1 μM tetrahydrodeoxycorticosterone (THDOC) exposure significantly increased tonic current amplitude. Pre-exposure to GFX prevented the 1 μM THDOC-mediated increase. *Significantly different to control (*p* < 0.05; *t*-test, *n* = 6–12 cells). **(B)** Examples of tonic currents from Fmr1 KO mice slices pre-incubated for 15 min with vehicle, or 100 nM SGE-516. Bottom. SGE-516 concentration-dependent increase in the tonic current amplitude. GFX prevented the 1 μM SGE-516-mediated increase. *Significantly different to control (*p* < 0.05; *t*-test, *n* = 6–12 cells).

We assessed the effects of NASs on tonic inhibition in hippocampal slices from *Fmr1 KO* mice. Exposure to 100 nM THDOC for 15 min followed by a >30 min washout period had small effects on tonic current, in contrast to this 1 μM THDOC induced a >3-fold increase in tonic current in *Fmr1 KO* animals (*p* = 0.0001, Figure 5A). THDOC treatment effectively restored tonic current to WT levels. Pre-exposure to the PKC inhibitor, GF109203X (GFX, 50 μM) prevented the 1 μM THDOC-mediated increase.

The limited bioavailability and short half-lives of endogenous NASs such as ALLO or THDOC or first generation synthetic NASs such as Ganaxolone has limited their development as therapeutics (Rupprecht, 2014). Because of these issues, we have examined a synthetic NAS from SAGE Therapeutics that has an improved pharmacokinetic profile, SGE-516, in hippocampal slices from *Fmr1 KO* mice. Hippocampal slices were exposed to SGE-516 for 15 min followed by a >30 min washout period before recordings were made from DGGCs. Exposure to SGE-516 caused a concentration-dependent reversal of the tonic current deficit with SGE-516 increasing tonic current from 20.6 ± 4.3 pA (*n* = 8) to 75 ± 13 pA with 100 nM SGE-516 (*n* = 5, *p* = 0.013) and 119 ± 13.5 pA with 1 μM SGE-516 (*n* = 7, *p* = 0.0004, Figure 5B). Pre-treating the hippocampal slices with GFX 15 min prior to SGE-516 treatment prevented the SGE-516-mediated enhancement of tonic current.

Contrary to the enhanced tonic current following 15-min NAS exposure, the same experimental protocol with hippocampal slices exposed to either THDOC or SGE-516 for 15 min resulted in no differences in amplitude, decay, or frequency of sIPSCs recoded from DGGCs from *Fmr1 KO* mice (data not shown). This data supports our earlier findings (Abramian et al., 2014; Modgil et al., 2017) that NAS preferentially increase the surface membrane expression of α4 subunit-containing extrasynaptic GABA_A_Rs in DGGCs, and furthermore, could be used to reverse the deficits in such receptors seen in Fragile X to enhance tonic inhibition.

### Increased Excitability of Granule Cells in *Fmr1 KO* Mice Is Reduced With NAS Treatment

**Figure 6:**
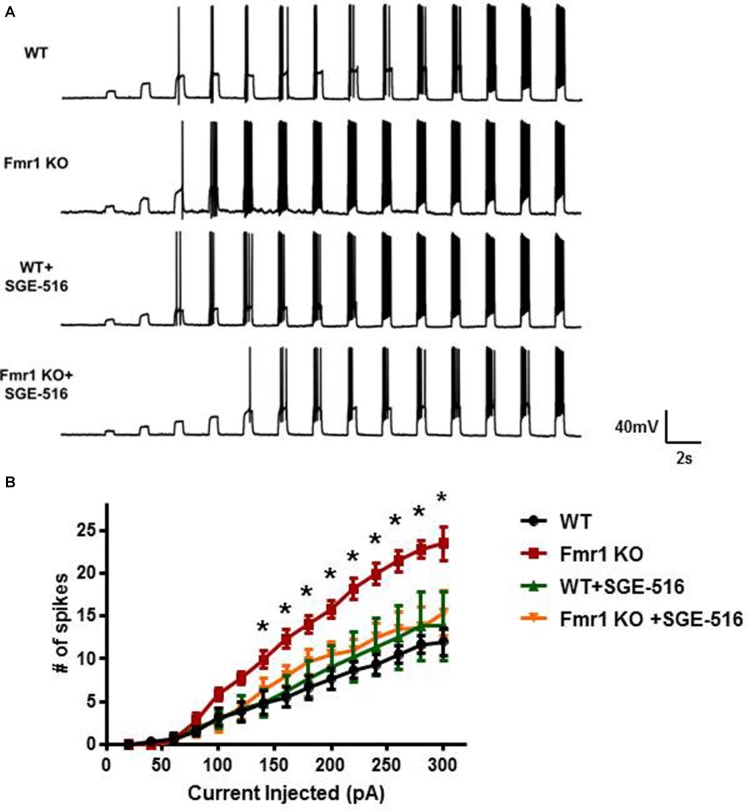
DGGCs from *Fmr1* *KO* mice are more excitable than from WT mice. **(A)** Representative recordings of action potential firing in DGGCs from WT and *Fmr1 KO* mice in response to 0.5 s current injections from 20 to 300 pA in 20 pA increments. **(B)** Average input/output curves representing the average number of action potential firing in DGGCs from WT and *Fmr1 KO* mice following exposure to vehicle or SGE-516 (100 nM). Values are means ± SEM; *n* = 3 mice, six cells per experimental group. *Denotes statistical significance of Fmr1 KO slice treated with 100 nM SGE-516 compared to vehicle treated Fmr1 KO slice using Student’s *t*-test for each current injection. For 160 pA *p* = 0.023, 180 pA *p* = 0.034, 200 pA *p* = 0.011, 220 pA *p* = 0.002, 240 pA *p* = 0.004, 260 pA *p* = 0.005, 280 pA *p* = 0.003, 300 pA *p* = 0.03.

Neuronal excitability is greatly influenced by tonic inhibition. Because of the decrease in DGGC tonic inhibition observed in *Fmr1 KO* mice we next examined input-output curves on DGGCs to measure the intrinsic excitability of the neurons and to see if NAS treatment could alter the excitability. We delivered current step injections to depolarize the membrane and measured the action potentials elicited from those current injections. Although there was no difference in resting membrane potential between DGGCs from WT and *Fmr1 KO* mice (WT −91.3 ± 3 mV, *n* = 6; *Fmr1 KO* −88 ± 2 mV, *n* = 7, *p* = 0.39), there was a clear significant increase in action potential firing rate in DGGCs from *Fmr1 KO* mice compared to WT DGGCs, with a leftward shift in the relationship between firing rate and injected current (Figure 6).

The ability of NASs to reverse the deficit in tonic current would suggest that NAS exposure could reduce neuronal excitability. Hippocampal slices were exposed to SGE-516 for 15 min followed by a >30 min washout period before the input-output relationship was examined. SGE-516 exposure significantly reduced membrane excitability of DGGCs from *Fmr1 KO* mice. There was no significant difference between *Fmr1 KO* DGGCs exposed to 100 nM SGE-516 to that of vehicle treated WT DGGCs (Figure 6). When WT hippocampal slices were exposed to SGE-516 there was no change in the action potential firing rate of DGGCs compared to vehicle treated controls. This lack of effect on action potential firing in WT suggests that the NAS-mediated increase in the level of tonic current through increased GABA_A_R levels in the surface membrane, as previously shown (Modgil et al., 2017), does not impact the excitability of the membrane.

Collectively our results demonstrate that there is a significant deficit in tonic current in *Fmr1 KO* mice leading to excessive neuronal excitation. This deficit in tonic current can be reversed by exposure to NASs to effectively restored tonic current and reduce excitation to wild-type levels.

## Discussion

In FXS, neuronal circuits are altered as shown by aberrant EEG activity (Van der Molen and Van der Molen, 2013). *Fmr1 KO* mice exhibit greater synchronized neuronal activity compared to the desynchronized activity in WT mice. The synchronized neuronal activity has been explained by an increase in action potential firing rates, particularly in Up states (Goncalves et al., 2013). The increased firing rates have been suggested to be a result of a deficit in GABAergic inhibition. Extrasynaptic α4 containing GABA_A_Rs are outward rectifying, meaning that the conductance based tonic inhibition, would be stronger at depolarized potentials and so are critical determinants in firing rates (Pavlov et al., 2009). Altered network activity that leads to circuit hyperexcitability has been proposed to underlie the neurological and psychiatric phenotypes of FXS (Deidda et al., 2014). Thus, manipulating the expression and/or activity of extrasynaptic GABA_A_R subunits may provide a therapeutic benefit in FXS.

NASs are positive allosteric modulators (PAMs) of GABA_A_Rs. The ability of NASs to potentiate both phasic and tonic inhibition makes them attractive anticonvulsants however despite these favorable properties the use of NASs as therapeutics has been hampered by their low bioavailability (Rupprecht, 2014). In addition to their actions as PAMs, NASs have profound effects on the expression levels of GABA_A_Rs, most notably those incorporating α4 subunits, effects that have previously been suggested to be dependent upon PKC activity (Leidenheimer and Chapell, 1997; Hodge et al., 2002; Harney et al., 2003; Koksma et al., 2003; Choi et al., 2008). Consistent with this, our recent studies have demonstrated that tetrahydrodeoxycorticosterone (THDOC), ALLO, and SGE-516 metabotropically increase the PKC-dependent phosphorylation of S443 in the α4 and S408/9 in β3 subunits. Enhanced phosphorylation of α4 and β3 subunits increases GABA_A_R insertion into the plasma membrane and reduces GABA_A_R endocytosis from the membrane, leading to a sustained increase in GABA_A_R tonic current density (Abramian et al., 2010, 2014; Adams et al., 2015). The potential clinical usefulness of NASs to treat some phenotypes of FXS has recently been shown in a recent pre-clinical study, it was demonstrated that SGE-516 was efficacious at reducing the audiogenic seizures in *Fmr1* KO mice (Hammond et al., 2017). But it remains to be seen if this anti-seizure property is mediated by the allosteric or metabotropic actions of SGE-516.

A possible reason why an increase in β3 S408/9 phosphorylation is detected in young *Fmr1 KO* mice is that protein phosphatase 2A (PP2A) is the phosphatase that mediates dephosphorylation of β3 S408/9 and FMRP. Not only is PP2A required for dephosphorylation of FMRP but PP2A and FMRP forms a synaptic signaling complex (Narayanan et al., 2007) which may be disrupted with the loss of FMRP and hence decrease PP2A activity and decrease dephosphorylation of β3 S408/9. The contribution that alterations in basal PP2A activity play in regulating S408/9 phosphorylation in *Fmr1 KO* mice remains to be assessed.

A recent immunogold labeling study observed a reduction of α4 subunits at perisynaptic and extrasynaptic sites and an increase in α4 subunits at synaptic sites suggesting that, in *Fmr1 KO* mice, the α4 subunit is mistrafficked into the synapse (Zhang et al., 2017). This electron microscopy finding by Zhang et al is in accordance with our results showing a change in benzodiazepine efficacy at synaptic GABA_A_Rs. Our data suggest an increased contribution of α4 subunit containing GABA_A_Rs to synaptic currents in DGGCs of *Fmr1* KO mice. The clinical usefulness of benzodiazepines in FXS is limited because of an ineffective response and high incidence of unwanted behavioral side effects (Marrosu et al., 1987; Tranfaglia, 2011, 2012). The association of α4 subunit containing GABA_A_Rs, which are not sensitive to benzodiazepines, to the synapse may provide a mechanism to explain the poor clinical efficacy of benzodiazepines in FXS.

The reduced tonic current recoded in *Fmr1 KO* DGGCs is not a result of reduced ambient GABA because in our recording solutions we added exogenous GABA. In addition, previous studies have noted no changes to the density or size of interneurons of the DG in *Fmr1 KO* mice suggesting the postsynaptic changes to phasic and tonic inhibition are not due to presynaptic changes (Selby et al., 2007).

In a recent clinical trial with the NAS ganaxolone in children and adolescents with FXS, no statistical significant improvement was observed for anxiety, attention, and hyperactivity (Ligsay et al., 2017). In our previous study, while a potent positive allosteric modulator, ganaxolone did not display any metabotropic activity (Modgil et al., 2017). This raises an interesting possibility that a portion of the therapeutic profile of NASs may be due to the metabotropic actions of NASs. The present study also noted changes in GABA_A_R subunit expression and phosphorylation were observed in younger *Fmr1 KO* mice and not in older animals. This would suggest that during a critical developmental period the alterations in GABA_A_Rs lead to long lasting developmental affects. Any therapeutic intervention directed towards GABA_A_Rs would need to be applied during this young age.

In conclusion, we have demonstrated an age-dependent alteration in expression and phosphorylation of GABA_A_R subunits of the hippocampus of *Fmr1 KO* mice. During this period there was a significant reduction in GABAergic tonic current in DGGCs and a drastic increase in neuronal excitation. A 15-min exposure to the NAS, SGE-516, effectively restored the tonic current and neuronal excitation back to WT levels. Excessive neuronal activity has been proposed to underlie the neurological and psychiatric phenotypes of FXS, thus, age-appropriate normalization of neuronal activity with a metabotropic NAS may have a significant therapeutic application.

## Data Availability

The datasets generated for this study are available on request to the corresponding author.

## Author Contributions

AM, TV, MA, JD, SM, and PD contributed to the conception and design of the study. AM, TV, and PD acquired, analyzed and interpreted data. AM and PD wrote the first draft of the manuscript. All authors contributed to manuscript revision, read and approved the submitted version.

## Conflict of Interest Statement

MA and JD are employed by SAGE Therapeutics. SM serves as a consultant for SAGE Therapeutics and AstraZeneca, relationships that are regulated by Tufts University. The remaining authors declare that the research was conducted in the absence of any commercial or financial relationships that could be construed as a potential conflict of interest.
